# Poorly Differentiated Neuroendocrine Tumor of the Rectum Coexistent with Giant Rectal Villous Adenoma Presenting as McKittrick-Wheelock Syndrome

**DOI:** 10.1155/2015/242760

**Published:** 2015-11-22

**Authors:** Sammy G. Nakhla, Traci T. Murakami, Srinath Sundararajan

**Affiliations:** ^1^Department of Medicine, Southern Arizona VA Health Care System, Tucson, AZ 85723, USA; ^2^Department of Gastroenterology, Southern Arizona VA Health Care System, Tucson, AZ 85724, USA; ^3^Department of Hematology/Oncology, University of Arizona Cancer Center, Tucson, AZ 85724, USA

## Abstract

McKittrick-Wheelock Syndrome is a rare disorder, noted for electrolyte and fluid depletion caused by secretory colorectal adenomas and carcinomas. We report here the first reported case of a 55-year-old man with a large rectal villous adenoma coexistent with a poorly differentiated neuroendocrine tumor of rectum presenting with McKittrick-Wheelock Syndrome. Palliative chemotherapy resulted in complete resolution of symptoms and improved quality of life.

## 1. Introduction

McKittrick-Wheelock Syndrome (MKWS) is a disorder with significant electrolyte and fluid derangement caused by a secretory colorectal tumor [1–4]. The clinical and laboratory features commonly seen in McKittrick-Wheelock Syndrome are mucus diarrhea, dehydration, hyponatremia, hypokalemia, prerenal azotemia, and metabolic acidosis [1, 3, 4]. Villous adenomas cause the majority of these cases [1]. Villous adenomas have higher risk for malignant transformation and sizes >2 cm increase risk significantly [5]. Rarely MKWS has been seen in patients with colorectal adenocarcinomas.

## 2. Case Report

A 55-year-old roofing consultant presented to the emergency department with complaints of chronic persistent diarrhea (6 stools/24 hours) in the last year. Laboratory tests revealed serum sodium (Na) level of 111 mmol/L, potassium (K) level of 3.1 mmol/L, blood urea nitrogen (BUN) level of 189 mg/dL, creatinine (Cr) level of 10.3 mg/L, and creatine phosphokinase (CPK) level of 12,700 IU/L. The peripheral leukocyte count was 17 × 10^3^ leukocytes/*μ*L, hemoglobin was 17 g/dL, and platelet count was 326 × 10^3^ platelets/*μ*L. Patient was hydrated with intravenous fluid replacement and placed on hemodialysis for acute renal failure and rhabdomyolysis. After intravenous fluid resuscitation and hemodialysis, physical and laboratory parameters normalized (Na, 136 mmol/L; K, 4.0 mmol/L; BUN, 17 mg/dL; Cr, 1.2 mg/dL, and CPK < 150 IU/L). Patient was discharged home after complete resolution of symptoms.

Within a few days, patient presented again to the emergency department with abdominal discomfort and intractable diarrhea. He was noted to have prerenal acute kidney injury with a serum creatinine of 1.8 mg/dL and leukocytosis. An extensive work-up to identify the etiology of diarrhea was performed. Stool tests including fecal fat, celiac disease serology, 24-hour urine indole-acetic acid, serum vasoactive intestinal peptide, and serum gastrin levels were normal.

**Figure 1:**
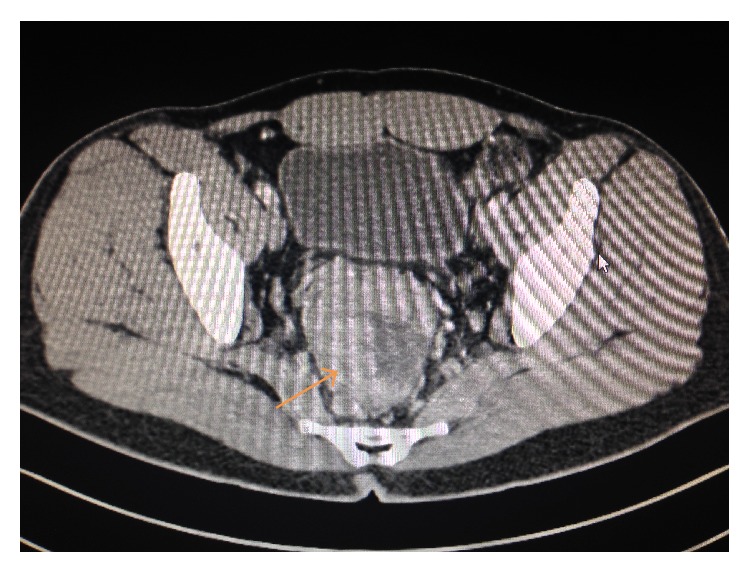
Abdominal CT scan showing large exophytic enhancing mass in the rectosigmoid colon with diffuse wall thickening.

**Figure 2:**
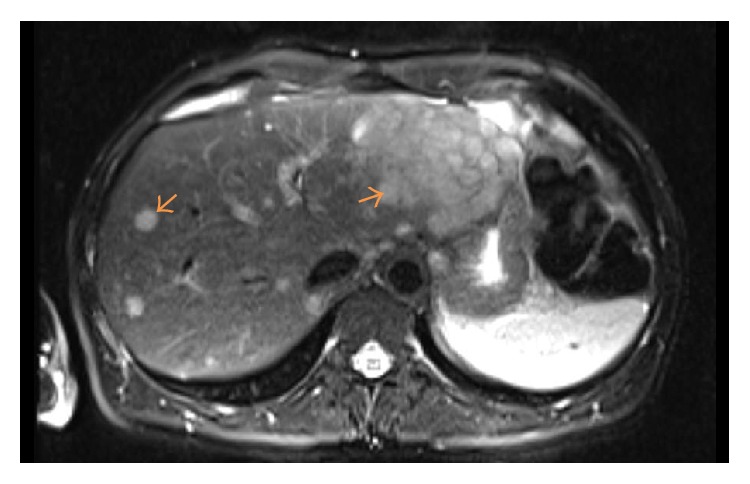
Abdominal MRI imaging scan showing a large dominant mass in the left hepatic lobe along with numerous other smaller hepatic lesions.

Computerized tomography (CT) scan of abdomen/pelvis revealed extensive circumferential rectal wall thickening measuring 13 cm in craniocaudal length with adjacent pelvic lymphadenopathy (Figure 1). Low attenuation hepatic lesions with the largest lesion in hepatic lobe measuring approximately 7.8 × 9.2 cm were also noted. Further imaging with magnetic resonance imaging (MRI) of the abdomen revealed innumerable T2 hyperintense slightly ill-defined heterogeneously enhancing metastases throughout the hepatic parenchyma, with a dominant mass in the lateral segment of left hepatic lobe measuring 10.4 cm in size (Figure 2).

**Figure 3:**
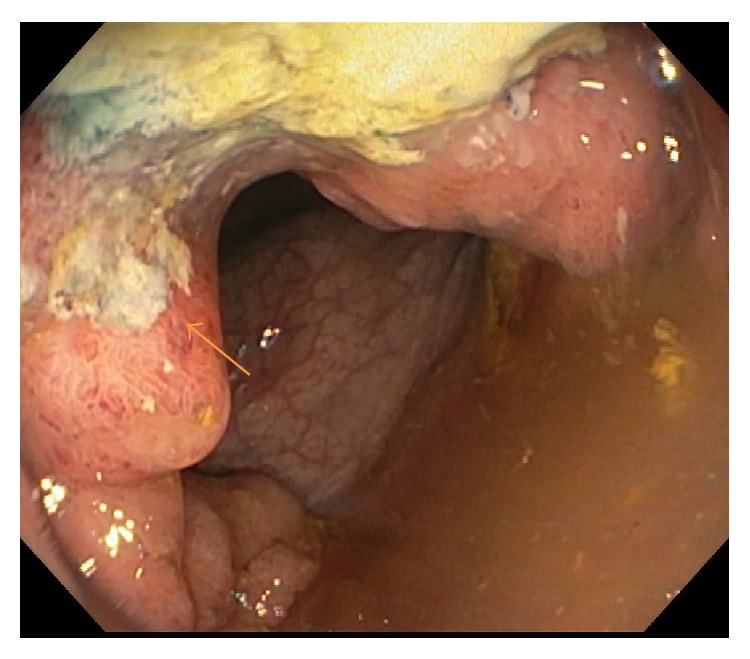
Colonoscopy showing friable ulcerated nearly obstructing mass at the anus extending up to 17-18 cm in the anal canal and rectosigmoid region.

**Figure 4:**
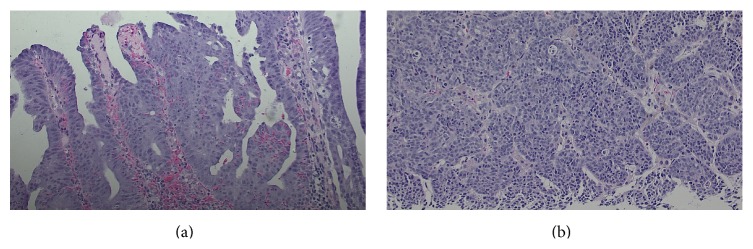
(a) Microscopic appearance of rectum mass biopsy revealing villous adenoma with focal high-grade dysplasia (H&E, ×20). (b) Microscopic appearance of liver mass biopsy revealing metastatic poorly differentiated neuroendocrine carcinoma of rectum (H&E, ×20).

**Figure 5:**
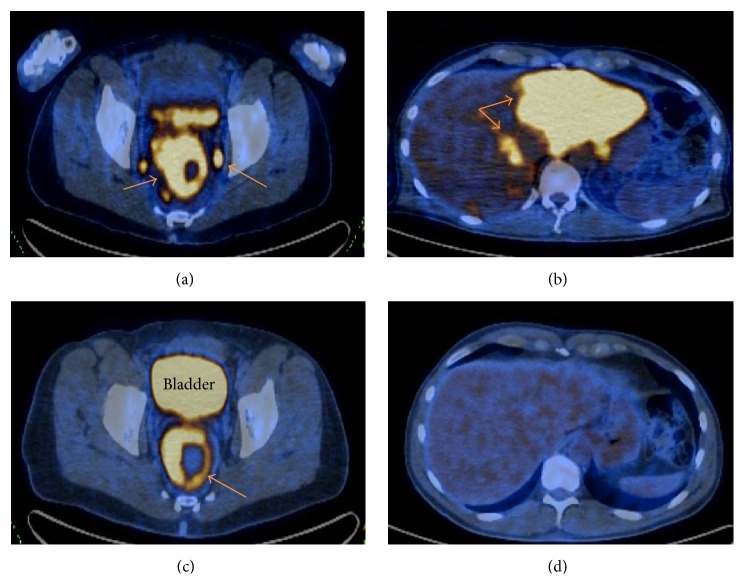
PET/CT scan showing intense FDG avid. (a) Rectal mass and surrounding lymphadenopathy. (b) Multiple liver lesions at diagnosis. (c) Interval partial response of the rectal mass to therapy. (d) Complete metabolic resolution of liver lesion and posttreatment changes noted after 6 cycles of chemotherapy.

Full colonoscopy demonstrated a friable semicircumferential mass at the anus extending up to 17-18 cm into the anal canal/rectosigmoid; the other parts of the colon were grossly unremarkable (Figure 3). Pathological report of the rectum mass biopsy revealed villous adenoma with focal high-grade dysplasia and necrosis. Invasive malignancy was not present in the biopsy fragments; however, adjacent invasive tumor could not be completely ruled out (Figure 4). A liver biopsy was recommended and pathological report of the liver mass biopsy revealed metastatic poorly differentiated neuroendocrine tumor (NET) of rectum (lesion stained positive for MAK 6 pankeratin, CDX2, and synaptophysin and negative for CK7, S100, and chromogranin) (Figure 5). Patient underwent a positron emission tomography (PET/CT) and a magnetic resonance imaging of the brain as part of his metastatic work-up. PET/CT (Figures 5(a) and 5(b)) work-up confirmed the 2-fluoro-2-deoxy-D-glucose (FDG) avid rectal mass with standardized uptake value (SUV) of 13.9 along with perirectal, pelvic lymph nodes, and multiple liver metastases (SUV of 16.4). No brain metastasis was noted. He was offered palliative systemic chemotherapy with carboplatin and etoposide for stage IV poorly differentiated NET of rectum (extrapulmonary small cell cancer). Patient was started on an octreotide injection to alleviate diarrhea symptoms. Patient had remarkable improvement of diarrhea, electrolyte derangements, and fatigue after one cycle of chemotherapy and was able to stop octreotide injections. After 6 cycles of chemotherapy, patient was noted to have a complete metabolic resolution of liver metastasis and partial response of primary rectal tumor (Figure 5). He has returned to his full time job as a roofing consultant.

## 3. Discussion

McKittrick-Wheelock Syndrome (MKWS) is a rare but serious complication of rectal adenomas [6]. The first case was described by McKittrick-Wheelock in 1954 of a secretory diarrhea associated with a villous adenoma [1]. The MKWS generally is a triad of acute renal failure, electrolyte abnormalities, and chronic diarrhea [7]. A complete colonoscopy is important in achieving the correct diagnosis.

Approximately 2% of cases with rectosigmoid villous adenomas will result in hypersecretory complications [8]. The typical rectal adenomas usually are greater than 4 cm but can range up to 18 cm at their greatest dimension [9]. These adenomas are primarily found in the rectum; however, cases have been reported in the sigmoid [1, 4, 10]. Larger adenomas and distally located tumor result in more severe laboratory and clinical symptoms. MKWS is postulated to be secondary to local secretagogue effect (mediated by prostaglandin E2 (PGE2)) of rectal adenoma. Rectal effluent in a case report demonstrated levels 3 to 6 times higher of PGE2 from a villous adenoma [9]. Hypersecretion can also result in severe azotemia along with electrolyte imbalances that could lead to cardiac arrhythmias and, in severe cases, rhabdomyolysis or altered mental status may be seen [11, 12].

This condition can be life threatening if secretant villous adenomas are left untreated [13]. Aggressive fluid resuscitation and electrolytes repletion are important especially prior to resection of the tumor. Endoscopic resection is the preferred treatment of choice; however, surgical resection is indicated for larger or circumferential adenomas with invasive cancer. Laboratory abnormalities usually improve after endoscopic resection of the rectal adenoma [14]. Nonreversible cyclooxygenase inhibitor indomethacin has been used to reduce PGE2 production, which consequently resulted in decreased loss of sodium and water through the rectum. Long-acting somatostatin analogue injection octreotide has also been shown to be effective in decreasing diarrhea symptoms especially in individuals contraindicated or intolerant of taking indomethacin [15].

Multiple cases of NET coexisting with adenomatous polyps have been reported before [16–18]. Considering the histology of liver lesion, we believe the patient likely had foci of NET in his rectal mass, which was not biopsied. Patients with poorly differentiated NET behave aggressively and are usually treated similar to extensive stage small cell lung cancer. In a study by Mitry et al. [19], poorly differentiated neuroendocrine tumors were chemosensitive to the cisplatin plus etoposide. Unfortunately, prognosis was poor with a 2-year survival rate approximately lower than 20% necessitating new therapeutic strategies to be developed.

## 4. Conclusion

To the best of our knowledge, this is the first reported case of rectal villous adenoma coexistent with a poorly differentiated NET of rectum presenting with McKittrick-Wheelock Syndrome. MKWS is a rare cause of severe hyponatremia and hypokalemia accompanied by watery diarrhea and fluid depletion as a result of hypersecretary rectal tumor. Early diagnosis and management of life threatening electrolyte and systemic abnormalities are essential. Surgical resection of the adenoma is definitive treatment of McKittrick-Wheelock Syndrome. McKittrick-Wheelock Syndrome associated with neuroendocrine tumor of rectum responds well to systemic chemotherapy.
